# Trait Procrastination and Mobile Phone Addiction Among Chinese College Students: A Moderated Mediation Model of Stress and Gender

**DOI:** 10.3389/fpsyg.2020.614660

**Published:** 2020-12-01

**Authors:** Xiaofan Yang, Pengcheng Wang, Ping Hu

**Affiliations:** Department of Psychology, Renmin University of China, Beijing, China

**Keywords:** college students, gender, stress, mobile phone addiction, trait procrastination

## Abstract

Recent studies have indicated that trait procrastination as a personality factor could lead to mobile phone addiction, however little is known about the mediating and moderating mechanisms underlying this process. The current study investigated the mediating role of stress in the relationship between trait procrastination and mobile phone addiction, and whether the mediating effect was moderated by gender. A sample including 1,004 Chinese college students completed measurements of trait procrastination, stress, mobile phone addiction, and demographic information. The results showed that trait procrastination was positively related to college students’ mobile phone addiction. Mediation analyses revealed that this relationship was partially mediated by stress. Moderated mediation further indicated that the path between trait procrastination and stress was stronger for male students compared with female students. These findings broadened our knowledge of the underlying mechanisms between trait procrastination and mobile phone addiction, the implications and limitations of this study were discussed.

## Introduction

In modern society, mobile phones have already become a necessity in our daily life. With continuously updated functions, especially as an important terminal of mobile internet, mobile phones can fulfill various demands including entertainment, life services, and access to information. According to a report from the China Internet Network Information Center, the number of mobile internet users in China had increased to 932 million by June 2020, accounting for 99.2% of the total internet users (CNNIC, 2020). Although mobile phones have greatly improved the convenience of people’s social life, mobile phone addiction is becoming a serious social problem that plagues a significant proportion of the population. Mobile phone addiction refers to the difficulties in daily life and loss of self-control due to the overuse of mobile phones (Cho et al., 2017). It was found to be related to a series of negative outcomes such as poor mental and physical health, academic failures, and interpersonal problems (Augner and Hacker, 2012; Lepp et al., 2014; Dwyer et al., 2018; Wang et al., 2019a). Therefore, it is particularly important for researchers to identify the potential risk factors that contribute to mobile phone addiction.

Among various factors related to mobile phone addiction, personality traits have been proven to be important predictors of mobile phone addiction. For instance, previous studies have found that impulsivity (Dewitte and Schouwenburg, 2002; Wu et al., 2013; Cerniglia et al., 2019), neuroticism (Billieux et al., 2015), trait anxiety (Mehroof and Griffiths, 2010; Hong et al., 2012; Billieux et al., 2015), and sensation seeking (Wang et al., 2019b) constituted risk factors of mobile phone addiction. Among these personality factors, trait procrastination has been found to be positively correlated with mobile phone addiction in recent years (Rozgonjuk et al., 2018; Wang et al., 2019a). However, the association between trait procrastination and mobile phone addiction needs to be examined in larger and more diverse samples. Besides, the underlying mechanism involved in this process is still largely unknown and thus remains to be further explored. Therefore, this study aims to address these gaps by testing the mediating role of stress and the moderating role of gender in the relationship between trait procrastination and mobile phone addiction in a sample of Chinese college students. This would provide us with a better understanding of the personality factor that influences mobile phone addiction, advance our knowledge of the emotional mechanism underlying this process, and provide new directions of how to prevent and reduce mobile phone addiction in male and female college students more effectively.

### Trait Procrastination and Mobile Phone Addiction

Procrastination is defined as “voluntarily delay of an intended action despite knowing to be worse off for the delay” (Steel, 2007). When it developed into a dispositional response to tasks that are perceived to be difficult, aversive, or lacking an immediate reward, procrastination can be viewed as a generalized personality trait (Steel and Ferrari, 2013; Sirois, 2014; Kroese and de Ridder, 2015; de Palo et al., 2017), and determines procrastination behaviors (Howell et al., 2006; de Palo et al., 2016).

Scholars generally believe that procrastination reflects a form of self-regulation failure (Ferrari, 2001; Sirois et al., 2003). From this point, lacking the self-control (Sirois and Pychyl, 2013) and goal-management ability (Gustavson et al., 2014) necessary for task engagement makes it difficult for procrastinators to inhibit their impulses and resist temptations. Thus, individuals who habitually procrastinate are more likely to indulge in immediate pleasure, meanwhile ignoring the pursuit of future goals (Sirois and Pychyl, 2013). Therefore, compared with tasks that are boring and (or) take effort, distractors characterized by immediate gratification are more attractive to procrastinators (Wang et al., 2019a). Mobile phones, in light of their powerful functions for entertainment and social contact, and their high accessibility can become an obsession for college students very easily.

From the perspective of personality determines behavior, we hold that procrastination as a personal characteristic could influence problematic mobile phone use. According to the “personality as predictor of dangerous behavior model” (Suls and Rittenhouse, 1990), certain personality traits that are typical of procrastinators, such as impulsivity and sensation seeking could lead to the selection and exposure to situations that elicit reactivity and create unnecessary stress (Gustavson et al., 2014; Wang et al., 2019a). Wang et al. (2019a) found that procrastination tendency can mediate the prediction of sensation seeking on mobile phone addiction. On one hand, individuals high in sensation seeking may be easily attracted by stimuli that provides high arousal; on the other hand, sensation seeking may act as the motivation of procrastination, which aims to experience the pleasure of high arousal when working against a deadline (Ferrari and Tice, 2000; Ferrari, 2001; Wang et al., 2019a). Therefore, it is reasonable for procrastinators to develop a behavior pattern of excessive use of mobile phones to satisfy these motives. Empirical evidence also showed that trait procrastination could positively predict problematic smartphone use (Rozgonjuk et al., 2018). In spite of this, this relation has rarely been examined in a sample of Chinese college students. So, we put forward the following hypothesis:

*Hypothesis 1*: Trait procrastination would positively predict mobile phone addiction among the Chinese college students.

### The Mediating Role of Stress

Stress is considered as an agitated state stemming from a lack of means to meet various social or environmental demands (DeLongis et al., 1988; Cho et al., 2017). Ample evidence has shown that stress is related to mental health problems such as emotional disorders (Maciejewski et al., 2000; Moeini et al., 2008; Stead et al., 2010; Pasca and Wagner, 2012), poor health conditions, and illness (Baldwin et al., 1997; Dougall and Baum, 2001). As a direct consequence of procrastination is the inability to complete a planned task on time, stress will emerge naturally due to the failure to meet internal and external expectations. For college students, chronic procrastinators might experience more stress due to their poor academic performance and related social comparisons with their peers. In addition, the direction of causality is worth noting. Although there is evidence indicating that procrastination could be an outcome of increased stress (Tice et al., 2001), this explanation of the direction of prediction will only make sense when procrastination is viewed as an act. From the perspective of characteristics, it is reasonable that the trait-like behavioral style of procrastination leads to the experience of stress rather than the reverse (Sirois, 2014). Moreover, negative automatic thoughts and rumination about past procrastination would further contribute to the maintenance of these negative emotions (Flett et al., 2012; Sirois, 2014). A stable correlation between trait procrastination and stress has been confirmed by a number of empirical studies (Sirois et al., 2003; Stead et al., 2010; Sirois, 2014). Longitudinal research further indicated that stress could be an outcome of procrastination (Sirois et al., 2009; Rice et al., 2012).

Moreover, empirical evidence indicated that stress is positively correlated with mobile phone addiction (Chiu, 2014; Jeon and Jang, 2014; Cho et al., 2017). Stress may lead to college students’ overuse of mobile phones for various reasons. First, there is a tendency for individuals to deal with their negative emotions through passive coping mechanisms such as substance abuse and addictive behaviors (Lapierre et al., 2012), thus with the increase of psychological stress, the problematic behavior of addiction may arise (DeLongis et al., 1988). When surrounded by negative mood, behaviors that could provide immediate reward seem to be the best choice to downregulate negative emotions. As the most convenient and accessible electronic equipment, mobile phones that can satisfy multiple needs instantly may easily become a readily available means to relieve stress (Cho et al., 2017; Fu et al., 2020; Monacis et al., 2020). Second, negative emotion can reduce an individual’s resistance to addictive temptations by weakening the ability of self-control (Cho et al., 2017) and inhibiting the activities of executive function (Eldar and Bar-Haim, 2010). Empirical study found that stress could predict smartphone addiction through reduced self-control (Cho et al., 2017).

Recently, an integrated framework called the “Interaction of Person-Affect-Cognition-Execution (I-PACE)” model was put forward to explain the development and maintenance of internet-use disorders (Brand et al., 2016; Young and Brand, 2017), and furthermore a series of addictive behaviors such as mobile phone addiction (Brand et al., 2019). According to this theory, specific internet-use disorders are believed to be the result of the interaction between predisposing variables, and mediating and moderating factors of internet-use disorders (Young and Brand, 2017). An individual’s core traits stemming from neurobiological and psychological construction will affect the subjective perception of the situation, then elicit corresponding emotional and cognitive responses, and further affect executive functions and the decision-making process (Brand et al., 2016). That is, trait procrastinators would experience more stress in their daily life. To cope with the stress, they are likely to develop a behavior pattern of mobile phone addiction as a way to relieve stress, although the situation may even get worse in the long run. Here, the second hypothesis was raised:

*Hypothesis 2*: Stress would play a mediating role between trait procrastination and mobile phone addiction.

### The Moderating Role of Gender

According to our hypothesis, trait procrastination may predict mobile phone addiction through the effect of stress, while the intensity of this mediation effect is unlikely to be identical for all the college students. We argue that gender may play a role in this process. To be specific, this study will test whether the link between trait procrastination and stress experienced by college students would be moderated by their gender.

It has been postulated that differences in sociality and autonomy will be reflected in one’s sensitivity to certain types of stressful life events (Beck, 1983; Verma et al., 2011). Studies demonstrated that women are more vulnerable to interpersonal problems, such as getting along with others in their proximal social network, or the loss of a confidant (Kendler et al., 2001; Verma et al., 2011). In contrast, men reported higher rates of events related to social performance as their source of stress, as typical stressors for males include job loss, legal problems, and work problems (Kendler et al., 2001). Physiological evidence further suggests that under psychosocial stressors related to standard performance (e.g., public speaking), stronger acute hypothalamic-pituitary-adrenal (HPA) and autonomic responses were found in men compared with women (Kajantie and Phillips, 2006). In short, it is reasonable to speculate that men will experience more stress when facing situations related to work or academic performance. Given that academic performance and other campus activities associated with social performance are often the top priorities for college students, male college students who habitually procrastinate may suffer more from stress caused by procrastination compared with their female peers.

Moreover, gender differences in emotion regulation could be an important reason why trait procrastination leads to more stress in male students. Prior research reflects that females are more likely to regulate their negative emotions than males (Fujita et al., 1991). In addition, men and women tend to employ different emotion regulation strategies to deal with negative emotions. Evidence showed that females are more active in seeking emotional support and prefer to use cognitive reappraisal to regulate negative emotions (Tamres et al., 2002), while males use expression suppression more frequently as their regulation strategy (Flynn et al., 2010). Considering that reappraisal is related to more effective regulation of negative emotions and less physiological activation under stress compared to suppression (Gross, 1998; Egloff et al., 2006), female students may be better at coping with their stress associated with procrastination than male students. To our knowledge no previous research has focused on the role of gender in the relationship between procrastination and stress, here we put forward the third hypothesis:

*Hypothesis 3*: Gender would moderate the relationship between trait procrastination and stress. Specifically, trait procrastination would result in more stress for male college students.

### The Present Study

**Figure 1:**
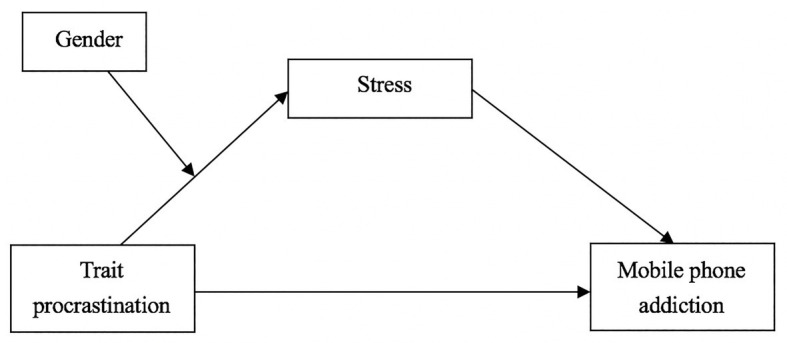
The proposed moderated mediation model.

Based on the literature review above, the present study constructed a moderated mediation model to examine the mediating role of stress in the relationship between trait procrastination and mobile phone addiction among Chinese college students. Furthermore, we tested whether the indirect path between trait procrastination and stress would be moderated by their gender. The proposed theoretical model is illustrated in Figure 1.

## Materials and Methods

### Participants

The participants were recruited from a college in Hebei Province, China. A total of 1,004 college students (234 of them were males) participate in this survey. The mean age was 19.40 years (*SD* = 1.44, range = 16–26 years, three data were missing). The participants completed a survey including demographic variables, trait procrastination, mobile phone addiction, and stress.

### Measures

#### Trait Procrastination

Following prior research (Sirois, 2014), we adopt the General Procrastination Scale (GPS; Lay, 1986) to measure students’ trait procrastination. The scale consists of 20 items (e.g., “I often find myself performing tasks that I had intended to do days before”). Participants rated each item on a 5-point scale ranging from 1 (strongly disagree) to 5 (strongly agree). All the items were averaged after 10 reversely scoring items were coded, with higher scores indicating higher trait procrastination. For the current study, Cronbach’s alpha for this scale was 0.81.

#### Stress

Stress was measured by the Stress Subscale of Depression Anxiety Stress Scale 21 (DASS-21; Lovibond and Lovibond, 1995) which consists of seven items (e.g., “I tend to over-react to situations”). Participants were required to select the response which best describes how much the statement applied to them over the past 2 weeks. Each item was scored on a 4-point scale, ranging from 0 (did not applied to me at all) to 3 (applied to me very much). A composite score was computed by averaging the seven items, with higher scores indicating the stronger experience of stress. In this study, Cronbach’s alpha was 0.85.

#### Mobile Phone Addiction

Mobile phone addiction was assessed by the Mobile Phone Addiction Index (MPAI; Leung, 2008). The instrument was comprised of 17 items, and a representative item was “You find yourself engaged on your mobile phone for a longer period of time than intended.” The measurement was based on a 5-point scale ranging from “1 = Never” to 5 = “Always,” and a composite score was calculated by averaging all the items. The higher the total scores, the more addicted the students were to their mobile phones. For the current study, Cronbach’s alpha for this scale was 0.89.

### Procedure

The study was approved by the research ethics committee of the first author’s institution. Informed consent was obtained from the students before the collection of data. Participants were told that their participation was entirely voluntary and that they could terminate at any time. The data collection was based on a project aiming at the investigation of college students’ mental health during October of 2019. Three teachers of the college were trained by the researcher to ensure the standardization of procedures. The survey was conducted online for all the participants through a survey software together with several other questionnaires.

### Statistical Analyses

First, descriptive information and correlational results of the variables were analyzed. Second, the mediating effect of stress was tested using Model 4 of the PROCESS macro for SPSS (Hayes, 2013). Third, we used Model 7 of the PROCESS macro to investigate the moderating role of gender on the mediation effect, i.e., whether the prediction of trait procrastination on stress was moderated by gender. All the continuous variables were standardized in modeling to eliminate the unit of measurement difference, so that the relative strength of different variables was comparable. Bootstrap confidence intervals (CIs) were applied to determine whether the effects in Model 4 and Model 7 were significant from 1,000 random samples of the data. CIs excluding zero indicated significant effects.

## Result

### Preliminary Analyses

Descriptive information and a correlation matrix of the variables are provided in Table 1. Correlation analysis showed that trait procrastination was positively related to mobile phone addiction (*r* = 0.40, *p* < 0.001), as well as stress (*r* = 0.29, *p* < 0.001). Stress was positively associated with mobile phone addiction (*r* = 0.44, *p* < 0.001).

**Table 1:** Descriptive statistics and correlations for all variables.

	*M*	*SD*	1	2	3
1.TP	2.71	0.51	1		
2.MPA	2.82	0.72	0.40^***^	1	
3.Str	1.07	0.65	0.29^***^	0.44^***^	1

*N* = 1,004. TP, trait procrastination; MPA, mobile phone addiction; Str, stress.

*** *p* < 0.001.

As the role of gender was one of the main concerns in this study, the descriptive information of the variables in the male and female groups are presented in Table 2. The *t*-test indicated that there were no significant gender differences in mobile phone addiction and trait procrastination, while male students reported more stress than female students, in the marginal significant level (*p* = 0.052).

**Table 2:** Descriptive information of the variables in the male and female groups.

	Gender	*N*	*M(SD)*	*t*	*p*
TP	Male	234	2.87 (0.71)	0.98	0.33
	Female	770	2.81 (0.73)		
MPA	Male	234	2.74 (0.71)	1.03	0.30
	Female	770	2.71 (0.73)		
Str	Male	234	2.15 (0.67)	1.95	0.05
	Female	770	2.05 (0.64)		

TP, trait procrastination; MPA, mobile phone addiction; Str, stress.

### The Mediating Role of Stress

The mediating role of stress in the relationship between trait procrastination and mobile phone addiction was tested utilizing Model 4 of the PROCESS macro. Controlling for age and grade as covariates, results showed that trait procrastination significantly predicted mobile phone addiction (*b* = 0.41, *p* < 0.001). After adding stress as a mediation variable, trait procrastination was still a significant predictor of mobile phone addiction (*b* = 0.30, *p* < 0.001). Moreover, trait procrastination positively predicted stress (*b* = 0.29, *p* < 0.001), and stress positively predicted mobile phone addiction (*b* = 0.35, *p* < 0.001), see Table 3 for details. In addition, the bias-corrected bootstrap test indicated a significant indirect effect of stress (*CI* = 0.078 to 0.137; effect size = 0.104), the mediation effect accounted for 25.72% of the total effect. Therefore, stress partially mediated the relationship between trait procrastination and mobile phone addiction.

**Table 3:** Testing the mediation model of stress.

Predictors	Model 1	Model 2	Model 3
	MPA	Str	MPA
	*b t*	*b t*	*b t*
TP	0.41 13.96^***^	0.29 9.74^***^	0.30 10.62^***^
Str			0.35 12.50^***^
*R^2^*	0.17	0.10	0.28
*F*	65.45^***^	35.12^***^	95.42^***^

*N* = 1,004. Each column is a regression model that predicts the criterion at the top of the column. The beta values are standardized coefficients. TP, trait procrastination; Str, stress; MPA, mobile phone addiction.

*** *p* < 0.001.

### The Moderated Mediation Model

Hypothesis 3 assumed that gender would moderate the indirect relationship between trait procrastination and mobile phone addiction *via* stress. To examine the moderated mediation hypothesis, we used the PROCESS macro (Model 7) to test the moderated mediation model. To be specific, the current study estimated the moderating effect of gender on the relationship between trait procrastination and college students’ stress. For detailed information on the models, see Table 4.

**Table 4:** Testing the moderated mediation effect of trait procrastination on mobile phone addiction.

Predictors	Model 1	Model 2
	Str	MPA
	*b t*	*b t*
TP	0.25 7.50^***^	0.30 10.62^***^
Gen	0.18 2.46^*^	
TP × Gen	0.20 2.65^**^	
Str		0.35 12.50^***^
*R^2^*	0.11	0.28
*F*	24.09^***^	95.42^***^

*N* = 1,004. The beta values are standardized coefficients. Gender is encoded as a dummy variable in the model: 1 = male, 0 = female. TP, trait procrastination; Gen, gender; Str, stress; MPA, mobile phone addiction.

* *p* < 0.05;

** *p* < 0.01;

*** *p* < 0.001.

**Figure 2:**
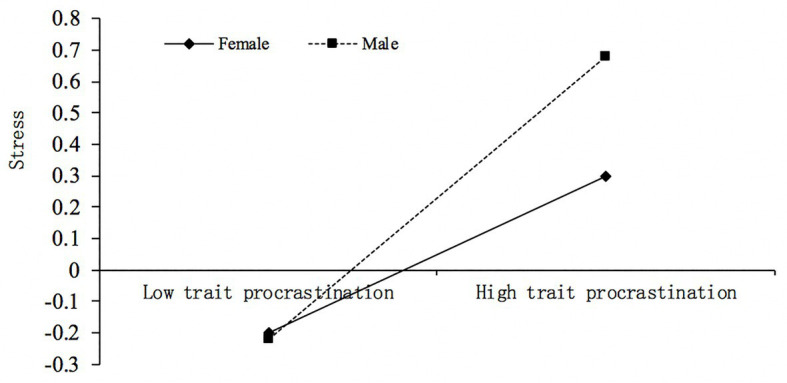
Gender moderates the relationship between trait procrastination and stress.

As Table 4 demonstrates, Model 1 indicated that there was a significant main effect of trait procrastination on stress, *b* = 0.25, *p* < 0.001, and just as we predicted, this effect was moderated by gender, *b* = 0.20, *p* < 0.01. For descriptive purposes, this study plotted the prediction of trait procrastination on stress, separately for male and female college students (Figure 2). Simple slope tests showed that the association between trait procrastination and stress was stronger for male college students (bsimple = 0.45, *p* < 0.001) than for female college students (bsimple = 0.25, *p* < 0.001).

In addition, the bias-corrected bootstrap analyses indicated that the indirect effect of trait procrastination on mobile phone addiction was moderated by the gender of the college students. For female students, the indirect effect of trait procrastination on mobile phone addiction was significant (*β* = 0.09, *SE* = 0.01, *CI* = 0.06–0.12). For male students, the indirect effect on mobile phone addiction was stronger (*β* = 0.16, *SE* = 0.03, *CI* = 0.11–0.22), as we predicted. Thus, hypothesis 3 was confirmed.

## Discussion

Although a number of studies have focused on the association between trait procrastination and mobile phone addiction, the potential mechanisms underlying the process remain to be explored. To this end, the current study proposed a moderated mediation model to test the role of stress and gender in the process. Results showed that stress played a mediating role between trait procrastination and mobile phone addiction, and this mediating effect was moderated by the gender of college students.

### Trait Procrastination and Mobile Phone Addiction

Consistent with our hypothesis, this study showed that procrastination, as a personality trait could be a risk factor for mobile phone addiction. Our findings not only confirmed that trait procrastination could positively predict mobile phone addiction as per a previous study (Rozgonjuk et al., 2018), but also extended this result to a sample of Chinese college students. We argue that this phenomenon could be explained by the underlying self-regulation difficulties shared by these two variables. Procrastinators, who are characterized by their low self-control and preference for short-term rewards, can easily become mobile phone addicts due to the designed features of the device. Besides, this study expanded the existing “personality - dangerous behavior” theory to the field of mobile phone addiction. As a typical maladaptive coping strategy (Kim and Seo, 2015), procrastination is supposed to be related to other dysfunctional behaviors such as drug or alcohol abuse (Sirois and Kitner, 2015). Our study further generalized previous works by demonstrating that trait procrastinators are also more likely to overuse mobile phones, which is also considered as a form of passive coping (Caplan, 2002).

### The Mediating Role of Stress

Furthermore, this study showed that stress partially mediated the relationship between trait procrastination and mobile phone addiction. Although quite a number of studies have supported the correlation between procrastination and stress (Sirois et al., 2009; Stead et al., 2010), as well as stress and mobile phone addiction (Jeon and Jang, 2014; Cho et al., 2017), to our knowledge, this study is the first to investigate the mediating role of stress between trait procrastination and mobile phone addiction.

Consistent with our assumption, trait procrastination could predict mobile phone addiction through the indirect effect of stress. On one hand, our results are in line with previous findings which suggest a link between stress and maladaptive behaviors (Sirois et al., 2009; Cho et al., 2017); On the other hand, this study advanced our understanding of how trait procrastination leads to mobile phone addiction. Our results supported the “I-PACE” model that specific personal characteristics will result in negative emotional reactions through the perception of the situation, and lead to certain addictive tendencies (Brand et al., 2016). As a result of weakened task orientation, chronic procrastinators who have difficulty in meeting social demands will experience more stress due to their poor performance. In order to alleviate stress, they are more likely to overuse mobile phones to escape from real life stressors, therefore increasing the possibility of mobile phone addiction. Moreover, it is reasonable to deduce that a dynamic and mutually reinforcing process exists. That is to say, not only are individuals with procrastination tendencies more likely to develop a behavior pattern of mobile phone addiction due to an increased experience of stress, but indulging in mobile phones will occupy individual’s time instead of doing their tasks (Wang et al., 2019a), reduce self-control, and destroy future time orientation, in turn leading to more procrastination.

### The Moderating Role of Gender

At last, an important aim of the study was to explore the moderating role of gender on the indirect link between trait procrastination and mobile phone addiction. Specifically, we examined whether the first path of the mediational pathway was moderated by gender. As we predicted, the relationship between trait procrastination and stress was stronger for male college students compared with their female peers (Figure 2). This finding was in line with the viewpoint that there are gender differences in sensitivity to different stressors (Verma et al., 2011). From the perspective of evolution, since the primary mission for males is to provide resources for their family, their capacity to obtain resources should be valued most by others, especially females in their social network. In the context of modern society, this evolution strategy is considered to be reflected in men’s working ability or public performance that are directly related to wealth acquisition. Therefore, poor working performance is expected to result in more stress in men than women, owing to its greater survival and social significance for males. For college students, trait procrastination will be largely reflected in their academic performance. As academic success is one of the most important life goals at this stage, and to a great extent determines the career prospects and social status after graduation, it is reasonable to assume that male college students will experience more stress from poor academic performance results due to trait procrastination. Besides, as mentioned above, the frequency and effectiveness of emotion regulation may also be a reason why the same degree of trait procrastination leads to less stress for female students.

Overall, this study firstly integrated gender as a moderator into the trait procrastination - mobile phone addiction model. On the theoretical level, it deepened our understanding of the individual differences in this process; as for the practical level, we offered new directions of how to prevent and reduce mobile phone addiction in male and female college students purposively.

### Implications and Limitations

In addition to the theoretical significance mentioned above, our study has some important practical implications. First, the results indicated that trait procrastination was an important risk factor for mobile phone addiction. Therefore, how to guide college students to reduce procrastination behavior and then overcome procrastination habits deserves special attention. On one hand, teachers and counselors should help students realize that procrastination can have a negative influence on their mental health and increase the potential possibility of problematic behavior. On the other, intervention strategies aimed at reducing procrastination such as making plans and the “implementation intentions” method can be applied to trait procrastinators. Second, since stress mediated the association between trait procrastination and mobile phone addiction, students should be instructed to use effective emotion regulation strategies to cope with stress, such as cognitive reappraisal or seeking emotional support instead of more passive ways. Third, the indirect effect of trait procrastination on mobile phone addiction through stress was stronger for male than female students, thus additional attention should be paid to male students. Teachers can remind male students to improve their insight into their own behaviors more frequently and guide them to adopt positive coping strategies to deal with stress.

Still, several limitations in the study should be taken into account. First, as a cross-sectional design was used in this study, we can not make any causal inference. The certain direction of our prediction is based on the view that when procrastination is conceived as a trait-like ongoing behavioral style, it is more plausible that trait procrastination would influence emotional experience and behaviors, rather than the inverse (Sirois, 2014). Therefore, longitudinal or experimental studies could be conducted to confirm the causal relationships in this study. Second, this study utilized a convenience sample, the representativeness of the sample is limited. Future studies could use random or stratified sampling to collect data in diverse groups. Besides, although the moderating role of gender was proven in our study, it is based on a female-biased sex ratio. Future research should examine whether the current results could be generalized to samples of balanced gender ratio.

## Conclusion

In sum, the present study reveals that trait procrastination can be a risk factor for college students’ mobile phone addiction. Moreover, this relationship could be mediated by stress. Besides, the indirect path between trait procrastination and mobile phone addiction was moderated by gender, with the effect between trait procrastination and stress being stronger for males compared with female college students.

## Data Availability Statement

The raw data supporting the conclusions of this article will be made available by the authors, without undue reservation.

## Ethics Statement

The studies involving human participants were reviewed and approved by Institutional Review Board of Department of Psychology, RUC. The patients/participants provided their written informed consent to participate in this study. Written informed consent to participate in this study was provided by the participants’ legal guardian/next of kin.

## Author Contributions

XY designed the study, collected and analyzed the data, and wrote the manuscript. PW and PH revised the manuscript. All authors contributed to the article and approved the submitted version.

### Conflict of Interest

The authors declare that the research was conducted in the absence of any commercial or financial relationships that could be construed as a potential conflict of interest.

## References

[ref1] Augner C. Hacker G. W. (2012). Associations between problematic mobile phone use and psychological parameters in young adults. Int. J. Public Health 57, 437–441. 10.1007/s00038-011-0234-z, PMID: 21290162

[ref2] Baldwin D. R. Harris S. M. Chambliss L. N. (1997). Stress and illness in adolescence: issues of race and gender. Adolescence 32, 839–853. PMID: 9426807

[ref3] Beck A. T. (1983). “Cognitive therapy of depression: new perspectives” in Treatment of depression: Old controversies and new approaches. eds. Clayton P. Barrett J. (New York: Raven press), 265–290.

[ref4] Billieux J. Maurage P. Lopez-Fernandez O. Kuss D. J. Griffiths M. D. (2015). Can disordered mobile phone use be considered a behavioral addiction? An update on current evidence and a comprehensive model for future research. Curr. Addict. Rep. 2, 156–162. 10.1007/s40429-015-0054-y

[ref5] Brand M. Wegmann E. Stark R. Müllere A. Wölflingf K. Robbins T. W.. (2019). The Interaction of Person-Affect-Cognition-Execution (I-PACE) model for addictive behaviors: update, generalization to addictive behaviors beyond internet-use disorders, and specification of the process character of addictive behaviors. Neurosci. Biobehav. Rev. 104, 1–10. 10.1016/j.neubiorev.2019.06.032, PMID: 31247240

[ref6] Brand M. Young K. S. Laier C. Wölfling K. Potenza M. N. (2016). Integrating psychological and neurobiological considerations regarding the development and maintenance of specific internet-use disorders: an Interaction of Person-Affect-Cognition-Execution (I-PACE) model. Neurosci. Biobehav. Rev. 71, 252–266. 10.1016/j.neubiorev.2016.08.033, PMID: 27590829

[ref7] Caplan S. E. (2002). Problematic internet use and psychosocial well-being: development of a theory-based cognitive–behavioral measurement instrument. Comput. Hum. Behav. 18, 553–575. 10.1016/S0747-5632(02)00004-3

[ref8] Cerniglia L. Griffiths M. D. Cimino S. de Palo V. Monacis L. Sinatra M.. (2019). A latent profile approach for the study of internet gaming disorder, social media addiction, and psychopathology in a normative sample of adolescents. Psychol. Res. Behav. Manag. 12, 651–659. 10.2147/PRBM.S211873, PMID: 31496849 PMC6698172

[ref9] Chiu S. I. (2014). The relationship between life stress and smartphone addiction on Taiwanese university student: a mediation model of learning self-efficacy and social self-efficacy. Comput. Hum. Behav. 34, 49–57. 10.1016/j.chb.2014.01.024

[ref10] Cho H. Y. Kim D. J. Park J. W. (2017). Stress and adult smartphone addiction: mediation by self-control, neuroticism, and extraversion. Stress Health 33, 624–630. 10.1002/smi.2749, PMID: 28332778

[ref11] CNNIC (2020). The 43st China statistical report on internet development. Available at: https://www.cnnic.net.cn/hlwfzyj/hlwxzbg/

[ref12] de Palo V. Limone P. Monacis L. (2016). Why university students procrastinate their academic tasks. Turkish Online Journal of Educational Technology, December Special Issue: 1366–1371.

[ref13] de Palo V. Monacis L. Miceli S. Sinatra M. Di Nuovo S. (2017). Decisional procrastination in academic settings: the role of metacognitions and learning strategies. Front. Psychol. 8:973. 10.3389/fpsyg.2017.00973, PMID: 28670292 PMC5472685

[ref14] DeLongis A. Folkman S. Lazarus R. S. (1988). The impact of daily stress on health and mood: psychological and social resources as mediators. J. Pers. Soc. Psychol. 54, 486–495. 10.1037/0022-3514.54.3.486, PMID: 3361420

[ref15] Dewitte S. Schouwenburg H. C. (2002). Procrastination, temptations, and incentives: the struggle between the present and the future in procrastinators and the punctual. Eur. J. Personal. 16, 469–489. 10.1002/per.461

[ref16] Dougall A. L. Baum A. (2001). “Stress, health and illness” in Handbook of health psychology. eds. Baum A. Reversion T. A. Singer J. E. (Mahwah, NJ: Lawrence Erlbaum), 321–337.

[ref17] Dwyer R. J. Kushlev K. Dunn E. W. (2018). Smartphone use undermines enjoyment of face-to-face social interactions. J. Exp. Soc. Psychol. 78, 233–239. 10.1016/j.jesp.2017.10.007

[ref18] Egloff B. Schmukle S. C. Burns L. R. Schwerdtfeger A. (2006). Spontaneous emotion regulation during evaluated speaking tasks: associations with negative affect, anxiety expression, memory, and physiological responding. Emotion 6, 356–366. 10.1037/1528-3542.6.3.356, PMID: 16938078

[ref19] Eldar S. Bar-Haim Y. (2010). Neural plasticity in response to attention training in anxiety. Psychol. Med. 40, 667–677. 10.1017/S0033291709990766, PMID: 19627649

[ref20] Ferrari J. R. (2001). Procrastination as self-regulation failure of performance: effects of cognitive load, self-awareness, and time limits on “working best under pressure.” Eur. J. Personal. 15, 391–406. 10.1002/per.413

[ref21] Ferrari J. R. Tice D. M. (2000). Procrastination as a self-handicap for men and women: a task-avoidance strategy in a laboratory setting. J. Res. Pers. 34, 73–83. 10.1006/jrpe.1999.2261

[ref22] Flett G. L. Stainton M. Hewitt P. Sherry S. Lay C. (2012). Procrastination automatic thoughts as a personality construct: an analysis of the procrastinatory cognitions inventory. J. Ration. Emot. Cogn. Behav. Ther. 30, 223–236. 10.1007/s10942-012-0150-z

[ref23] Flynn J. J. Hollenstein T. Mackey A. (2010). The effect of suppressing and not accepting emotions on depressive symptoms: is suppression different for men and women? Personal. Individ. Differ. 49, 582–586. 10.1016/j.paid.2010.05.022

[ref24] Fu L. Wang P. Zhao M. Xie X. Chen Y. Nie J. (2020). Can emotion regulation difficulty lead to adolescent problematic smartphone use? A moderated mediation model of depression and perceived social support. Child Youth Serv. Rev. 108:104660. 10.1016/j.childyouth.2019.104660

[ref25] Fujita F. Diener E. Sandvik E. (1991). Gender differences in negative affect and well-being: the case for emotional intensity. J. Pers. Soc. Psychol. 61, 427–434. 10.1037/0022-3514.61.3.427, PMID: 1941513

[ref26] Gross J. J. (1998). Antecedent- and response-focused emotion regulation: divergent consequences for experience, expression, and physiology. J. Pers. Soc. Psychol. 74, 224–237. 10.1037/0022-3514.74.1.224, PMID: 9457784

[ref27] Gustavson D. E. Miyake A. Hewitt J. K. Friedman N. P. (2014). Genetic relations among procrastination, impulsivity, and goal-management ability: implications for the evolutionary origin of procrastination. Psychol. Sci. 25, 1178–1188. 10.1177/0956797614526260, PMID: 24705635 PMC4185275

[ref28] Hayes A. F. (2013). Introduction to mediation, moderation, and conditional process analysis: A regression-based approach. New York: Guilford Press.

[ref29] Hong F. Y. Chiu S. I. Huang D. H. (2012). A model of the relationship between psychological characteristics, smartphone addiction and use of mobile phones by Taiwanese female university students. Comput. Hum. Behav. 28, 2152–2159. 10.1016/j.chb.2012.06.020

[ref30] Howell A. J. Watson D. C. Powell R. A. Buro K. (2006). Academic procrastination: the pattern and correlates of behavioural postponement. Personal. Individ. Differ. 40, 1519–1530. 10.1016/j.paid.2005.11.023

[ref31] Jeon H. S. Jang S. O. (2014). Effects of stress and depression on smartphone addiction in college students: focus on management efficiency by gender. Korean J. Youth Stud. 21, 103–129.

[ref32] Kajantie E. Phillips D. I. (2006). The effects of sex and hormonal status on the physiological response to acute psychosocial stress. Psychoneuroendocrinology 31, 151–178. 10.1016/j.psyneuen.2005.07.002, PMID: 16139959

[ref33] Kendler K. S. Thornton L. M. Prescott C. A. (2001). Gender differences in the rates of exposure to stressful life events and sensitivity to their depressogenic effects. Am. J. Psychiatr. 158, 587–593. 10.1176/appi.ajp.158.4.587, PMID: 11282693

[ref34] Kim K. R. Seo E. H. (2015). The relationship between procrastination and academic performance: a meta-analysis. Personal. Individ. Differ. 82, 26–33. 10.1016/j.paid.2015.02.038

[ref35] Kroese F. M. de Ridder D. T. D. (2015). Health behavior procrastination: a novel reasoned route towards self-regulatory failure. Health Psychol. Rev. 10, 313–325. 10.1080/17437199.2015.1116019, PMID: 26530737

[ref36] Lapierre L. M. Hammer L. B. Truxillo D. M. Murphy L. A. (2012). Family interference with work and workplace cognitive failure: the mitigating role of recovery experiences. J. Vocat. Behav. 81, 227–235. 10.1016/j.jvb.2012.07.007

[ref37] Lay C. H. (1986). At last, my research article on procrastination. J. Res. Pers. 20, 474–495. 10.1016/0092-6566(86)90127-3

[ref38] Lepp A. Barkley J. E. Karpinski A. C. (2014). The relationship between cell phone use, academic performance, anxiety, and satisfaction with life in college students. Comput. Hum. Behav. 31, 343–350. 10.1016/j.chb.2013.10.049

[ref39] Leung L. (2008). Linking psychological attributes to addiction and improper use of the mobile phone among adolescents in Hong Kong. J. Child. Media. 2 93–113. 10.1080/17482790802078565

[ref40] Lovibond P. F. Lovibond S. H. (1995). The structure of negative emotional states: comparison of the Depression Anxiety Stress Scales (DASS) with the beck depression and anxiety inventories. Behav. Res. Ther. 33, 335–343. 10.1016/0005-7967(94)00075-u, PMID: 7726811

[ref41] Maciejewski P. K. Prigerson H. G. Mazure C. M. (2000). Self-efficacy as *A. mediator* between stressful life events and depressive symptoms: differences based on history of prior depression. Br. J. Psychiatry 176, 373–378. 10.1192/bjp.176.4.373, PMID: 10827887

[ref42] Mehroof M. Griffiths M. D. (2010). Online gaming addiction: the role of sensation seeking, self-control, neuroticism, aggression, state anxiety, and trait anxiety. Cyberpsychol. Behav. Soc. Netw. 13, 313–316. 10.1089/cyber.2009.0229, PMID: 20557251

[ref43] Moeini B. Shafii F. Hidarnia A. Babaii G. R. Birashk B. Allahverdipour H. (2008). Perceived stress, self-efficacy and its relations to psychological wellbeing status in Iranian male high school students. Soc. Behav. Personal. Int. J. 36, 257–266. 10.2224/sbp.2008.36.2.257

[ref44] Monacis L. Griffiths M. D. Limone P. Sinatra M. Servidio R. (2020). Selfitis behavior: assessing the Italian version of the selfitis behavior scale and its mediating role in the relationship of dark traits with social media addiction. Int. J. Environ. Res. Public Health 17, 1–17. 10.3390/ijerph17165738, PMID: 32784419 PMC7460134

[ref45] Pasca R. Wagner S. L. (2012). Occupational stress, mental health and satisfaction in the Canadian multicultural workplace. Soc. Indic. Res. 109, 377–393. 10.1007/s11205-011-9907-5

[ref46] Rice K. G. Richardson C. M. E. Clark D. (2012). Perfectionism, procrastination, and psychological distress. J. Couns. Psychol. 59, 288–302. 10.1037/a0026643, PMID: 22352949

[ref47] Rozgonjuk D. Kattago M. Täht K. (2018). Social media use in lectures mediates the relationship between procrastination and problematic smartphone use. Comput. Hum. Behav. 89, 191–198. 10.1016/j.chb.2018.08.003

[ref48] Sirois F. M. (2014). Procrastination and stress: exploring the role of self-compassion. Self Identity 13, 128–145. 10.1080/15298868.2013.763404

[ref49] Sirois F. M. Kitner R. (2015). Less adaptive or more maladaptive? A meta-analytic investigation of procrastination and coping. Eur. J. Personal. 29, 433–444. 10.1002/per.1985

[ref50] Sirois F. M. Melia-Gordon M. L. Pychyl T. A. (2003). “I’ll look after my health, later”: an investigation of procrastination and health. Personal. Individ. Differ. 35, 1167–1184. 10.1016/S0191-8869(02)00326-4

[ref51] Sirois F. M. Pychyl T. A. (2013). Procrastination and the priority of short-term mood regulation: consequences for future self. Soc. Personal. Psychol. Compass 7, 115–127. 10.1111/spc3.12011

[ref52] Sirois F. M. Voth J. Pychyl T. A. (2009). “I’ll look after my health, later”: A prospective study of the linkages of procrastination to health and well-being in undergraduate students. *Paper presented at the 6th Biennial conference of Counselling the Procrastinator in Academic Settings*. (Toronto, ON: York University).

[ref53] Stead R. Shanahan M. J. Neufeld R. W. J. (2010). “I’ll go to therapy, eventually”: procrastination, stress and mental health. Personal. Individ. Differ. 49, 175–180. 10.1016/j.paid.2010.03.028

[ref54] Steel P. (2007). The nature of procrastination: a meta-analytic and theoretical review of quintessential self-regulatory failure. Psychol. Bull. 133, 65–94. 10.1037/0033-2909.133.1.65, PMID: 17201571

[ref55] Steel P. Ferrari J. (2013). Sex, education and procrastination: an epidemiological study of procrastination characteristics from a global sample. Eur. J. Personal. 27, 51–58. 10.1002/per.1851

[ref56] Suls J. Rittenhouse J. D. (1990). “Models of linkages between personality and disease” in Personality and disease. ed. Friedman H. S. (New York: Wiley), 38–63.

[ref57] Tamres L. K. Janicki D. Helgeson V. S. (2002). Sex differences in coping behavior: a meta-analytic review and an examination of relative coping. Personal. Soc. Psychol. Rev. 6, 2–30. 10.1207/s15327957pspr0601_1

[ref58] Tice D. M. Bratslavsky E. Baumeister R. F. (2001). Emotional distress regulation takes precedence over impulse control: if you feel bad, do it! J. Pers. Soc. Psychol. 80, 53–67. 10.1037/0022-3514.80.1.53, PMID: 11195891

[ref59] Verma R. Balhara Y. P. S. Gupta C. S. (2011). Gender differences in stress response: role of developmental and biological determinants. Ind. Psychiatry J. 20, 4–10. 10.4103/0972-6748.98407, PMID: 22969173 PMC3425245

[ref60] Wang P. Liu S. Zhao M. Yang X. Zhang G. Chu X. (2019a). How is problematic smartphone use related to adolescent depression? A moderated mediation analysis. Child Youth Serv. Rev. 104:104384. 10.1016/j.childyouth.2019.104384

[ref61] Wang J. Wang P. Yang X. Zhang G. Wang X. Zhao F. (2019b). Fear of missing out and procrastination as mediators between sensation seeking and adolescent smartphone addiction. Int. J. Ment. Heal. Addict. 17, 1049–1062. 10.1007/s11469-019-00106-0

[ref62] Wu A. M. Cheung V. I. Ku L. Hung E. P. (2013). Psychological risk factors of addiction to social networking sites among Chinese smartphone users. J. Behav. Addict. 2, 160–166. 10.1556/JBA.2.2013.006 25215198 PMC4117295

[ref63] Young K. S. Brand M. (2017). Merging theoretical models and therapy approaches in the context of internet gaming disorder: a personal perspective. Front. Psychol. 8:1853. 10.3389/fpsyg.2017.01853, PMID: 29104555 PMC5655004

